# Healthcare needs and priorities of older people living with heart failure and frailty: a multi-perspective study of patients, carers and clinicians

**DOI:** 10.1186/s12877-025-06926-1

**Published:** 2026-01-07

**Authors:** Sunanthiny Krishnan, Mayuri Gogoi, Carolyn Tarrant, Simon Conroy, Louise Clayton, Iain B. Squire, Shirley Sze

**Affiliations:** 1 NIHR Cardiovascular Research Centre and Department of Cardiovascular Sciences, University of Leicester, Glenfield Hospital, Leicester, LE3 9QP UK; 2 Department of Respiratory Sciences, University of Leicester, Leicester, LE1 7RH UK; 3 Development Centre for Population Health, University of Leicester, Leicester, LE1 7RH UK; 4 Department of Population Health Sciences, University of Leicester, Leicester, LE1 7RH UK; 5 NIHR Greater Manchester Patient Safety Research Collaboration (GM PSRC), University of Manchester, Manchester, M13 9PL UK; 6 Barts Health NHS Trust and Queen Mary University of London, London, E1 4NS UK; 7 Department of Cardiology, Glenfield Hospital, Leicester, LE3 9QP UK

**Keywords:** Older people, Heart failure, Frailty, Healthcare needs, Health-related goals

## Abstract

**Background:**

Older people living with heart failure (HF) and frailty are a complex population growing in prevalence. Given the high level of comorbidity among these patients, a person-centered, holistic model of care is required to optimise outcomes in this cohort. However, there is limited knowledge on what matters to these patients and if current care pathways address their needs. We aimed to explore the healthcare needs and priorities of older people with HF and frailty from the perspective of patients, carers and clinicians.

**Methods:**

In a step-wise, multi-method study, we conducted a qualitative survey followed by in-depth interviews among older adults (≥ 65 years) with HF and frailty (Clinical Frailty Score ≥ 5), their informal carers (≥ 18 years) and healthcare professionals at a tertiary hospital in the UK. An inductive thematic analysis was performed on the data using the Framework Method.

**Results:**

Between January – May 2023, 160 individuals completed the survey and 23 participated in interviews. Combined analysis of surveys and interviews revealed two major themes (i.e., healthcare needs and perceived health-related goals) and 14 subthemes. Healthcare needs identified were: (i) management of medical issues; (ii) regaining physical functioning; (iii) pharmaceutical care; (iv) nutritional care; (v) assistance with activities of daily living (ADL); (vi) environmental and social support and (vii) access to healthcare. Their health-related goals were: (i) being ADL-independent; (ii) remaining HF-symptom free; (iii); improved quality of life; (iv) being with family; (v) not a burden to family; (vi) avoiding hospitalisation and (vii) longevity.

**Conclusion:**

Healthcare needs of older patients living with HF and frailty are manifold; a key finding was that preserving functional capacity was given greater importance than longevity by this population. A multidisciplinary approach aligned with patients’ health priorities is essential for delivery of a meaningful goal-concordant care.

**Supplementary Information:**

The online version contains supplementary material available at 10.1186/s12877-025-06926-1.

## Background

Heart failure (HF) is predominantly a disease of older people, with a mean age at diagnosis of 77 years [1]. A progressive, life-limiting condition, prevalence of HF increases steeply with age, from 1% among those aged less than 55 to more than 10% in those over 70 years of age [1, 2]. Frailty, a syndrome more common but not synonymous with ageing, is particularly common in older people with HF owing to overlapping pathophysiological mechanisms [3]. Patients with HF are up to six times more likely to be frail and conversely, frailty is associated with increased risk of HF, especially among older people [4, 5]. The presence of frailty in patients with HF is associated with adverse clinical outcomes, including recurrent and prolonged hospitalisations and increased risk of mortality [6, 7]. Longer hospital stay predisposes older patients to hospital- associated deconditioning which in turn accelerates functional decline, fuelling the vicious cycle of frailty [8].

Management of HF in older people is complex, due to the physiological deficits of ageing and the presence of comorbidities [9]. Current clinical guidelines are largely focused on single-disease management which often leads to high treatment burden and attendant healthcare consumption [10, 11]. Yet the evidence-base for improving outcomes for older people consistently points towards the need for a holistic approach [12]. It seems likely then that a frailty-attuned, multidisciplinary approach will help in managing older patients with HF and frailty [2]. Equally pivotal is to adopt person-centred care (PCC) within which patient’s individual needs and priorities are integrated into treatment plans, enabling them to take an active role in their own health [13]. Such a model of care has been shown to reduce symptom burden with improved clinical outcomes and better quality of life (QoL), and is associated with meaningful reduction in hospitalisation and utilisation of healthcare resources [14].

With the global population ageing, it is imperative for healthcare systems to be equipped with the knowledge to manage older people sustainably. Despite their prominence in the clinical setting, older patients living with HF and frailty remain a poorly represented population in research, owing to the complexity of their illness. There is a lack of understanding of what issues matter most to this patient population and if current healthcare pathways are adequate in addressing their needs.

### Study aim

In this study, we sought to explore the specific healthcare needs and health priorities of older people with HF and frailty from the perspective of patients, carers and healthcare professionals (HCPs). A thorough understanding of their needs and goals is necessary to bridge the knowledge gap and to inform delivery of a patient-centred care to optimise outcomes.

## Methods

### Study design

We designed a study to understand the healthcare needs of older people living with HF and frailty. In addition to patients living with these conditions, their informal carers as well as clinicians directly involved in their care were also engaged to gain a holistic perspective of the subject matter. Family members are instrumental in the delivery of long-term care for older patients, especially those with complex needs. They are closely, and often consistently, involved in addressing the care needs of their loved ones in their day-to-day lives. Given the long-standing relationship, informal carers serve as a vital source of collateral history [15] with in-depth knowledge of their care recipients’ needs, preference and capacity [16]. Similarly, frontline clinicians are expected to offer complementary insights into the needs of this patient population from their professional experience of caring for them in the hospital setting.

We utilised a step-wise approach of qualitative survey followed by interviews in order to elicit a breadth of responses and a rich dataset to enable deeper understanding of the complex care needs of this population. Methods and results of this study are presented in accordance with the Standards for Reporting Qualitative Research (SRQR) [17].

### Sampling and recruitment

We employed purposive, maximum variation sampling [18] to recruit older patients, their carers and clinicians in a single, tertiary National Health Service (NHS) hospital located in Leicestershire, one of the UK’s most ethnically diverse regions. Inclusion criteria for patients were: aged 65 and older with confirmed diagnosis of HF and Clinical Frailty Scale (CFS) score of 5 or more. The CFS is a frailty assessment tool that quantifies frailty on a 9-point scale (1- very fit to 9- terminally ill) [19]. The frailty score was determined by the researcher at the time of recruitment. Patients with evidence of cognitive impairment and those unable to provide informed consent were excluded. Informal carers (older than 18 years) who accompanied patients on their medical visit, and clinicians involved in the care of patients with HF were also invited to partake in the study. Recruitment took place at the outpatient HF clinic and inpatient cardiac wards.

### Data collection

#### Qualitative survey

A semi-structured questionnaire was used to elicit responses from patients to the following three open- ended questions: (1) *What are your main healthcare needs?* (2) *Regarding your health status*,* what matters most to you?* and (3) *Which area of healthcare would you like more support with?*. Carers and clinicians were asked analogous questions regarding healthcare needs and what they believed mattered most to older patients living with HF and frailty. The questionnaire was developed based broadly on the classic concept of ‘*what matters to you*?’ [13, 20, 21], with additional questions in line with the objective of the study. The questionnaire was reviewed by a panel of experts comprising a geriatrician, a HF consultant and a qualitative researcher. Participants were given a survey form to provide their responses in writing. The survey was administered in-person by two researchers, SS (a cardiologist specialising in HF and trained in qualitative methods) and SK (a clinical pharmacist with experience in qualitative research). Neither researcher was engaged in direct care of the patients at the time of study. For patients and carers with reduced dexterity, the researchers supported survey completion by transcribing verbal responses on to the survey form without the use of any video or audio devices. The responses were not recorded verbatim but paraphrased by the researchers. For participants with a language barrier, an interpreter was engaged to facilitate communication. The interpreter translated the questions and responses verbatim and the researchers documented the answers on the survey forms.

#### Interview

Following completion of the survey, the research team (SS and SK) undertook interviews with an additional sample of patients, carers and clinicians to provide depth to findings from the qualitative survey. A semi-structured interview guide was used adaptively to explore the subject in depth, drawing on the lived experiences of the study participants. The interview guide was developed in collaboration with a patient public involvement group consisting of ten patients with HF. The guide was constructed based on the framework proposed by Kallio et al. [22], adopting similar structure for all three groups of participants. The key topics explored include: (1) *What is the most valued aspect of health for patients with HF and frailty; what matters to these patients and why?* (2) *What are the healthcare needs of older patients with HF and frailty;* (3) *Does current HF services adequately address their needs? If not*,* what are the unmet healthcare needs?* Face validity of the guide was determined by a panel of experts (i.e., a geriatrician, a HF consultant and a qualitative researcher). All interviews were conducted face-to-face, in English and audio recorded. Recordings were transcribed verbatim by SK and cross validated by SS. Transcripts were not returned to participants for review.

### Data analysis

We adopted the Framework Method as delineated by Gale et al. [23] to analyse thematically the data from the surveys and interviews [24]. The analytical process began with the survey dataset whereby an inductive approach was used to code data and identify patterns [25]. Two researchers, SS and SK carried out the data analysis. Initially, the researchers independently read and re-read five entries from each participant group for familiarisation and identification of key concepts through open coding. This was followed by a collaborative deliberation of the entries and review of the open codes. A coding scheme was then developed jointly to serve as a working analytical framework, which was applied to remaining survey entries using Microsoft Excel by SK, and checked for accuracy by SS. The Framework was refined iteratively during coding. Responses from the various parts of the survey were treated as one cohesive dataset and coding was undertaken across the whole entry. Recruitment, survey administration and data analysis occurred simultaneously until saturation was reached and no new codes emerged from the survey data [26]. Interview transcripts were subsequently coded line-by-line, applying the analytical framework developed from the survey data. The framework was reorganised as needed based on new insights from the interviews. Both researchers coded all transcripts jointly to ensure consistency and interpreted the charted data synchronously to formulate overarching themes. Coding of interview dataset was managed using NVivo version 20.6.1.1137 (Lumivero).

### Quality

The research team established trustworthiness of the study by adapting the four salient criteria posited by Lincoln and Guba [27]. Credibility and dependability of our findings were ensured via three forms of triangulation (i.e., data, methods and investigator triangulation). The study was conducted across three distinct cohorts of participants to gather comprehensive insights into the subject matter. In addition to surveys, interviews were conducted to provide depth to the former. Two researchers were involved in data collection and analysis. An independent experienced qualitative researcher (MG) reviewed the analytical process to ensure qualitative rigor. Prolonged and insightful engagement with the data by the researchers throughout the study period further rendered itself to the credibility of data.

Transferability was ensured through researchers maintaining field notes during data collection (both surveys and interviews) affording meaningful interpretation of the responses within the context that they were gathered. An audit trail of raw data, transcripts, code generation and coding schema was retained to reinforce confirmability of the study findings. Throughout the study, the researchers were cognizant of their positionality and were mindful not to allow their own perspectives and experiences as HCPs overshadow those of the participants’. During data analysis, the team continually evaluated their personal perceptions to minimise any inherent bias in interpreting the data.

### Ethical consideration

This study was conducted according to the guidelines of the Declaration of Helsinki and approved by the Research Ethics Committee of Health Research Authority and Health Care Research Wales (REC Ref: 22/EM/0172). Written informed consent was obtained from all participants prior to the survey and interview.

This work was supported by the British Heart Foundation, UK (Grant Number: HFHF_025).

## Results

Table 1 represents the demographic characteristics of study participants. A total of 183 participants participated between January – May 2023. One-hundred and sixty participants completed the survey (68 patients, 41 carers, 51 clinicians) and 23 participated in the interview (10 patients, 5 carers and 8 clinicians). Each survey took 15 to 30 min to complete, whilst interviews lasted 45–60 min.

**Table 1:** Demographics of study participants

Characteristics	Surveys (*N* = 160)			Interviews (*N* = 23)		
	Patients (*n* = 68)	Carers (*n* = 41)	Clinicians (*n* = 51)	Patients (*n* = 10)	Carers (*n* = 5)	Clinicians (*n* = 8)
Age, year						
*Median (range)*	78 (65–92)	*NA*	*NA*	80 (69–90)	*NA*	*NA*
* 20–29*	*NA*	1 (2.4%)	29 (56.9%)	*NA*	*NA*	1 (12.5%)
* 30–39*	*NA*	9 (22.0%)	11 (21.6%)	*NA*	1 (20%)	4 (50.0%)
* 40–49*	*NA*	10 (24.4%)	6 (11.8%)	*NA*	*NA*	1 (12.5%)
* 50–59*	*NA*	7 (17.1%)	4 (7.8%)	*NA*	1 (20%)	2 (25.0%)
* 60–69*	8 (11.8%)	2 (4.9%)	1 (2.0%)	1 (10%)	1 (20%)	*NA*
* 70–79*	30 (44.1%)	4 (9.8%)	*NA*	4 (40%)	1 (20%)	*NA*
* 80–89*	29 (42.6%)	8 (19.5%)	*NA*	4 (40%)	1 (20%)	*NA*
* 90–99*	1 (1.5%)	*NA*	*NA*	1 (10%)	*NA*	*NA*
Gender						
* Male*	38 (55.9%)	15 (36.6%)	12 (23.5%)	8 (80%)	3 (60%)	2 (25.0%)
* Female*	30 (44.1%)	26 (63.4%)	39 (76.5%)	2 (20%)	2 (40%)	6 (75.0%)
Ethnicity						
* Caucasian*	41 (60.3%)	19 (46.3%)	21 (41.2%)	7 (70%)	2 (40.0%)	6 (75.0%)
* South Asian*	27 (39.7%)	22 (53.7%)	25 (49.0%)	3 (30%)	3 (60.0%)	1 (12.5%)
* Black*	*NA*	*NA*	2 (3.9%)	*NA*	*NA*	1 (12.5%)
* Arab*	*NA*	*NA*	1 (2.0%)	*NA*	*NA*	*NA*
* Mixed*	*NA*	*NA*	2 (3.9%)	*NA*	*NA*	*NA*
Clinical Frailty Score (CFS)						
* 5*	44 (64.7%)	*NA*	*NA*	5 (50%)	*NA*	*NA*
* 6*	22 (32.4%)			4 (40%)		
* 7*	2 (2.9%)			1 (10%)		
LVEF Phenotype						
* HFrEF*	31 (45.6%)	*NA*	*NA*	3 (30%)		
* HFmrEF*	17 (25.0%)			4 (40%)	*NA*	*NA*
* HFpEF*	20 (29.4%)			3 (30%)		
No. of Comorbidities						
* 2–3*	14 (20.6%)	*NA*	*NA*	*NA*	*NA*	*NA*
* 4–5*	27 (39.7%)			6 (60%)		
* 6–7*	15 (22.1%)			4 (40%)		
* 8–9*	9 (13.2%)			*NA*		
* ≥ 10*	3 (4.4%)			*NA*		
Occupation						
* Doctor*	*NA*	*NA*	24 (47.1%)	*NA*	*NA*	3 (37.5%)
* Nurse*			14 (27.5%)			3 (37.5%)
* Pharmacist*			4 (7.8%)			1 (12.5%)
* Dietitian*			*NA*			1 (12.5%)
* Physiotherapist*			2 (3.9%)			*NA*
* Occupational Therapist*			5 (9.8%)			*NA*
* Healthcare Assistant*			2 (3.9%)			*NA*

*LVEF* Left Ventricular Ejection Fraction, *HFrEF* Heart Failure with Reduced Ejection Fraction, *HFmrEF* Heart Failure with Mildly Reduced Ejection Fraction, *HFpEF* Heart Failure with Preserved Ejection Fraction

Median age of the patient-cohort was 79; 59% were male; 44% had heart failure with reduced ejection fraction (HFrEF). Two thirds (63%) of patients were mildly frail, 33% moderately frail, 4% severely frail. A significant proportion of study participants (38–54%) were of South Asian ethnicity, in keeping with the cultural diversity of the local population. Participants within the carer-cohort were essentially family members (i.e., spouses and adult children) providing informal care. The clinician-sample was comprised of various HCPs including doctors, pharmacists, nurses, dietitian, physiotherapists and occupational therapists. A geriatrician and a palliative care doctor were also engaged for the interviews.

**Fig. 1:**
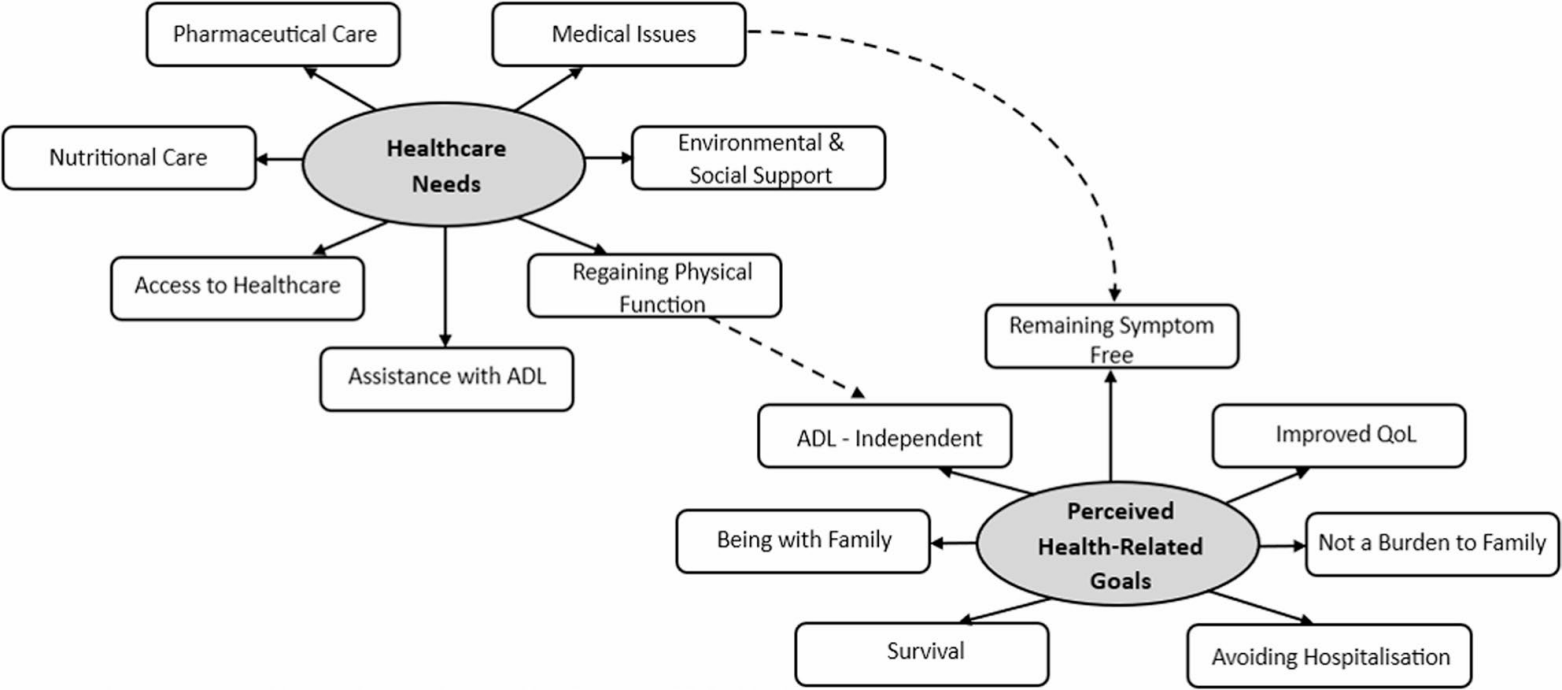
Overview of key themes and subthemes (Note: Solid lines represent direct links between themes and subthemes; dotted lines represent mediating effects between themes)

From the combined analysis of surveys and interviews, our study identified 2 major themes and 14 subthemes. The key themes were: *healthcare needs* and *perceived health-related goals* (Fig. 1).

### Theme 1: Healthcare needs: adopting a holistic outlook

The health needs of older patients with frailty and HF were distilled into seven subthemes, integrating patient- and service-related needs. Specific aspects of care within each healthcare need identified are presented in Table 2. Illustrative quotes of each subtheme according to participant groups are provided in Table S1 (Appendix).

**Table 2:** Specific aspects of healthcare needs of older patients living with heart failure and frailty

	Healthcare Needs	Specific Aspects of Care Need
*Patient- related*	Management of medical issues	*Management of HF symptom* *Management of co-morbidities* *Advance care planning*
	Regain physical functioning	*Able to get around* *Strength & balance* *Fall prevention* *Rehabilitation programme*
	Pharmaceutical care	*Pill burden* *Non-adherence* *Adverse drug reactions* *Education about medication*
	Nutritional care	*Poor appetite* *Education about HF- healthy diet*
*Service- related*	Assistance with ADL	*Personal care* *House chores* *Meal preparation* *Formal carer*
	Environmental & social support	*Home adaptation* *Psychosocial wellbeing*
	Access to healthcare	*Regular follow up* *Primary/ community-based care* *Secondary care* *Point-of-contact*

### Management of medical issues

For a vast majority of older patients, *inadequate control of their HF symptoms* was the most pressing issue at the time of the study. Shortness of breath, peripheral oedema and fatigue were reported frequently as debilitating symptoms affecting activities of daily living (ADLs) and mobility. For many, a walk to the toilet at home was a struggle. Carers and clinicians also emphasised the importance of adequate symptom management (particularly breathlessness) so that patients can “*go about their lives and enjoy their hobbies*” (doctor, female).

Aside from HF, almost half of the patient-participants expressed the need to have their *co-morbidities managed well at the same time*. Arthritis, musculoskeletal pain and respiratory problems were frequently stated as inadequately addressed. Carers specifically pointed out the importance of adopting a “*holistic approach*” (carer, female) when managing older patients, addressing all of their medical problems comprehensively.

Clinicians exclusively raised the concept of *advance care planning (ACP)* for these patients, highlighting the importance of having early conversations about prognosis and incorporating patients’ wishes into their care plan, whilst also acknowledging the difficulty in broaching the subject with patients and their families. They also advocated for timely referral to palliative care for optimisation of symptom management.

### Regain physical functioning

Restricted mobility was a source of frustration for patients and carers as it remarkably diminished their independence and QoL. Underlying causes were multifactorial: inadequate control of HF symptoms, frailty and musculoskeletal comorbidities among others. Patients wished they were *able to get around* more, and do things that they used to enjoy such as going on walks, gardening, travelling and engaging in sports. Regaining physical *strength and balance* was another important health need for many patients. They mentioned feeling “*fragile*” (patient, male); that they are “*unable to do anything without feeling strenuous*” (patient, male). Many experienced recurrent falls due to “*weak legs*” (patient, male) and “*loss of balance*” (patient, male). As a result, they “*lost confidence to walk*” (patient, male), and some were “*petrified of falling*” (carer, female) and therefore “*prefer to use a wheelchair*” (patient, male). All three participant groups highlighted *fall prevention* to be a significant health need in older people with HF. Clinicians emphasised on judicious use of medications such as diuretics and those that lower blood pressure, as they contribute to risk of falls and subsequent fragility fractures. A large proportion of clinicians also stated referral to *rehabilitation programmes* would be beneficial to build patients’ strength and confidence to mobilise, thereby improving their independence. Interestingly, three inpatient participants pointed out that the lack of physical activity on hospital wards significantly impacted their physical functioning post-discharge. Hospital-associated deconditioning was also heavily stressed by the clinicians, that could potentially be addressed by institution of inpatient rehabilitation programme.

### Pharmaceutical care

Among our patient-participants, *pill burden* was invariably a subject of discontentment. They “*get fed up with the medications as there were too many tablets*” (patient, male). On average, patients took around 15 tablets a day; one patient reported taking 40 pills daily for multiple health conditions. Clinicians and carers also recognised polypharmacy as a problem in this patient population, albeit to a lesser extent. A majority of our patients managed their medications using dosette boxes. As carers noted, these patients “*have difficulty remembering to take medications*” (carer, female). However, despite the use of compliance aids, some admitted to *non-adherence to medications*, especially to diuretic therapy due to increased urinary frequency, affecting not only their day-to-day living but also their social lives. Other *adverse effects of medications* that were of concern in this population include “*dizziness*” (patient, male) and anticholinergic burden. A few patients reported recurrent falls due to low blood pressure secondary to medications.

Clinicians, particularly doctors and nurses, highlighted the need to address continence needs associated at least in part with the use of diuretics, noting the risk of urinary tract infections and “*skin integrity issues due to moisture damage*” (nurse, female). Understanding about medications was generally poor among patients. A substantial proportion of carers fed back that more *education about medications* and side effects should be in place for both patients and carers. Clinicians noted that “*lots of patients do not seem to know what medications they are on for HF and why*” (doctor, female). Whilst optimisation of guideline- directed medical therapy (GDMT) for HF is a key priority, clinicians emphatically called for rationalisation of medications in this population, weighing the risks against the benefits of the treatment.

### Nutritional care

*Management of poor appetite* and weight loss was expressed as an important health need by a significant number of carers and patients. Interestingly, aside from the dietitian, not many clinicians recognised this as a problem. Some patients mentioned “*eating less due to breathlessness*” (patient, male), others reported issues with chewing and swallowing. Two participants were prescribed oral supplement to meet their daily nutritional needs. None of the other participants had ever had a dietetic review. Most of our patient-participants who lived alone relied on ready-made or frozen meals from supermarkets, for convenience. Generally, nutritional awareness was low among patients and carers (e.g., salt intake and colour coding on food labels). Unsurprisingly, clinicians indicated that *education about HF- healthy diet* was an important component of disease management.

### Assistance with ADL

Many of our patient-participants required help with their ADLs, particularly with *personal care*, *house chores* and *daily meal preparation*. Personal care needs spanned assistance with washing, toileting and dressing. The majority of patients were reliant on their family members to cater for their daily needs. A female patient who lived alone, could only “*have a bath once a week”* (patient, female) when her daughter was able to come around to help. Spousal carers in the study expressed their struggle with the role, often in the context of their own poor health status.

Working adults were limited in their caregiving responsibilities due to work commitments and needed more support for their frail parents especially when they are home alone. A significant degree of caregiver burden was observed (implied or expressed explicitly). Clinicians acknowledged that older patients with HF and frailty require considerable assistance with their ADLs and that care-needs assessment should be incorporated in their management plan with appropriate provision of social care services, especially for those living alone.

### Environmental and social support

A large number of our study patients required *home adaptations* to support their mobility and to remain independent. Installation of handrails around the house, walk-in showers and toilet aids were some of the frequently mentioned modifications. A few patients had sustained falls in the toilet because “*there was nothing to hold onto*” (patient, male). Several patients also wished to have stairlifts fitted as climbing stairs had become very strenuous. Some of these patients literally “*crawl their way up*” (patient, female) stopping often to catch their breath. Aids (such as walking frames and wheelchairs) were also frequently requested to help with mobility indoors and out. Clinicians duly recognised the need for home adaptation to ensure safety in frail patients. Prevention of pressure sores was also highlighted by the clinicians as a vital aspect of care in this group of patients, due to long hours of physical inactivity.

Physical health aside, a vast majority of the participants emphasised the importance of maintaining *psychosocial wellbeing* in older people with HF and frailty. A strong call to address their emotional and mental health was noted across all subgroups of participants. Carers particularly asked for more empathy and compassion from HCPs when caring for their frail older adults. Patients valued good family support, lack of which visibly had an emotional toll on some. A few battled with loneliness although they were co-residing with their adult children. Many felt staying connected via community support groups was not only important for its social values, but also enabled better understanding of their condition through interacting with other patients with similar problems and experiences.

### Access to healthcare

Timely access to healthcare, particularly *primary care*, was one of the most commonly cited service-related needs of patients with frailty and HF. Difficulties in getting general practitioners’ (GP) appointments and hour-long waits with the telephone booking system often led to delayed follow-up. A patient mentioned feeling extremely frustrated having had to “*wait for 3 weeks to see his GP”* (patient, male) for his breathlessness. Carers felt having *regular follow-ups* would enable better monitoring of patients’ progress and treatment response, which could also help alleviate caregiver stress as they were constantly “*concerned about the patient’s condition*” (carer, male).

Clinicians opined that regular follow up at the *community level* (by GP or community HF nurses) could prevent unnecessary hospitalisation. Several carers requested having a key worker as a *point-of-contact* to help coordinate care and minimize treatment burden for patients as well as themselves. A single, permanent point-of-contact could serve as an anchor of support and act as the first port of call when patients’ medical condition warrants escalation of management. The key worker could also help co-ordinate care between primary and *secondary care* specialties.

### Theme 2: Perceived health-related goals: beyond the hard metrics

Our study identified seven health-related goals that were perceived significant for older patients living with frailty and HF (Fig. 1).

Maintaining physical capacity to *manage ADLs independently* emerged as the utmost health priority for this group of patients. Participants of all subgroups equally felt strongly about improving the level of functional independence of older adults as it would help restore their “*dignity*” (doctor, male) and “*self-respect*” (carer, female); “*I want to improve my strength so I can be independent*” (patient, male). Staying independent would also give “*confidence to (their spouse)*” (patient, male) which patients valued as an important aspect in their lives.

Indeed, for patients living with HF, leading a comfortable life *free of symptoms* and having *a good QoL* was a resounding priority. Symptom burden and apprehensions about worsening symptoms especially, shortness of breath, often restricted many from engaging in tasks that they enjoyed, leaving them leading a precarious life. Clinicians stated that QoL should be the “*focus of decision making*” (doctor, female) in this cohort of patients, more than “*quantity of life*” (doctor, female).

*Spending quality time with loved ones* was quite prominent in patients’ response - “*I want to enjoy the rest of my life see my children…grandchildren…family*” (patient, female). For spousal carers, being around their partners was also equally important. A lone carer in his late 80s, would visit his wife every day in the hospital because he needed his company and *“she’s my company”* (carer, male). The concept was less apparent within the clinician-group, as only two participants highlighted it.

Prolonging *survival* did not come through as a significant priority in this patient population, with a fair level of agreement across the various participant groups. Whilst clinicians and carers regarded *avoidance of hospitalisation* as one of the top healthcare goals for this cohort of patients, it was, comparatively, less important for the patients. A considerable number of older patients spontaneously raised the concept of burden and wished they could be less of a *burden to their families*. These respondents were dependent on their family members for support with ADLs. Table S2 (Appendix) details exemplary quotes of each subtheme according to participant groups.

## Discussion

Older people with frailty constitute the majority of patients that physicians manage in HF clinics. However, this patient group is often excluded from clinical trials and their voices are not often heard in their own medical management. Previous works have explored the perception of frailty in HF patients, although little work has been done to understand the healthcare needs and goals of these patients. Su et al. [28] interviewed 13 patients with HF and frailty, and found that HF patients had limited knowledge about frailty; they also had difficulty acknowledging the presence of frailty. Liu et al. [29] studied the perception, knowledge and attitudes regarding frailty, from the perspective of HF patients and HCPs. The authors found that frailty was often perceived as a state of predicament, associated with feelings of weakness, reduced self-care abilities and depressive emotions. Frailty is often misunderstood as equivalent to “end of life”; the causes and potential reversibility of frailty were not recognised, leading to suboptimal management and poor outcomes in this vulnerable population. Whilst both studies highlight an important knowledge gap regarding frailty in HF populations, it is unclear how management strategies for this vulnerable population could be optimised. Our study addresses this key question by understanding the healthcare needs and goals of older people with HF and frailty, reflecting on existing HF pathways and identifying strategies to improve care delivery.

### Healthcare needs

Resolution of HF symptoms was the foremost response received from all study participants, particularly the patients with regards to their healthcare needs. Patients with HF experience significant symptom burden [30–32], comparable to that seen in patients with advanced malignancy [33]. This reflects the incapacitating impact HF symptoms have on patients’ daily functioning. Even with optimal therapy, patients experience increased symptom burden with disease progression over time [34].

With HF being a disease of unpredictable trajectory [30], ease of access to appropriate specialist services, including palliative care seems to be prudent, as highlighted by the clinician participants in the study. However, current lack of clear pathway for referral, as noted by our clinicians, means that palliative care is often not accessed until the patient is in the advanced stage of illness, depriving them of adequate symptom palliation and the autonomy for shared-decision making. Notwithstanding, ACP can be facilitated by any treating physician; this should be part of the routine consultation and not the sole responsibility of the palliative care team. However, as our study participants reflected, ACP discussions are sensitive and thus challenging. There is, potentially, an educational need on the subject in order to increase the uptake in practice.

Suboptimal management of patients’ comorbidities was highlighted in the study with an undertone of siloed care. Fragmentation of care is a major concern in older people living with multimorbidity and has been associated with increased emergency department visits and poor health outcomes [11, 35]. The narratives from our study participants evidenced their frustrations, especially over the gridlock in accessing healthcare services. It is important to note that a substantial proportion of hospitalisation and death in HF patients with frailty is due to non-cardiovascular causes [36]. Better management of comorbidities, adopting a whole person approach, could lead to better healthcare and clinical outcomes for this vulnerable patient group.

The issue of polypharmacy and the resultant pill burden could be attributed, at least in part, to the splintered care that patients received for their various comorbidities. Polypharmacy is associated with non-adherence and increased exposure to adverse drug reactions, and importantly is associated with increased risk of fall [37], an intrinsic vulnerability in older people living with frailty. Structured medication review with safe deprescribing within an integrated care may help alleviate patients’ medication burden. With concurrent education about medications and side effect profiles, adherence to therapy could also be improved.

Restoring physical capacities of older people with HF and frailty relates strongly to their health goals of functional independence and better QoL. The restriction in mobility in this patient population was due to a mixture of reduced effort tolerance due to HF, suboptimal symptom management and attrition in strength and balance associated with advanced age and frailty. History of falls, and the subsequent fear of falling, also crippled patients’ confidence to mobilise [38]. Given the heightened morbidity, a thorough assessment of fall risk should be made routine in the management of people with frailty. Referral to exercise-based cardiac rehabilitation would also benefit this patient population [39]. An individualised programme tailored to their physical status could improve their functional capacity and help maintain independence.

Home adaptation to complement older people’s physical competence is also integral for independent living [40]. In our study, patients with poorer frailty status needed more modifications to their immediate environment, and required greater support with their ADLs. In many cases, family members (i.e., spouses or adult children) assumed the responsibility of care, with some experiencing caregiver burden. Whilst some patients were already in receipt of package of care from NHS, this was often inadequate to meet their needs. Comprehensive assessments followed by periodic reviews of patients’ home environment and coping strategies are therefore warranted to ensure they remain supported with their day-to-day tasks.

Poor appetite was reported frequently by carers and patients in the study, particularly among those who were moderate-severely frail. Of note, few clinicians noted this as a problem. Anorexia of ageing is a significant cause of malnutrition in older adults which in turn accelerates frailty, but is seldom addressed in clinical practice [41]. The poor awareness of HF-healthy diet among patients and carers was also a cause for concern, given the potential adverse effect of dietary indiscretion on HF control. Comprehensive nutritional care that involves assessment and education is a clear necessity in this patient cohort.

The concept of psychological wellbeing resonated throughout the study. Evidence suggests that frailty is strongly associated with depressive symptoms in older adults [42]. A once active and independent individual, to now rely on others for core human needs such as eating, bathing and toileting could conceivably impact self-esteem and adversely affect mental health [43]. Social isolation due to reduced mobility may also be contributory. The desire expressed by our patient participants for social interactions and meaningful relationships indicates the important roles of family and support groups in maintaining the psychosocial wellbeing of older adults. Engagement with social groups could also increase patients’ knowledge about their medical conditions and enhance their coping management [44].

### Perceived health- related goals

Preservation of functional capacity emerged collectively as the most important health-related goal for older patients living with HF and frailty. This finding concurs with mounting evidence that older people value functional independence as a significant goal of care beyond the typical disease-specific metrics [45–47], corroborating calls by cardiovascular societies to prioritise functional capacity as a principal end point in managing older adults with cardiovascular disease [48]. Given the considerable effects of HF symptoms on physical function, adequate control of symptoms was, unsurprisingly, a crucial priority for our patient-participants, as was improved QoL over longevity and hospitalisation. Previous studies report similar preferences in outcome among patients with HF [32, 49]. On the contrary, avoidance of hospitalisation was raised as an important health-related goal by clinicians, understandably so as recurrent HF hospitalisation is associated with adverse prognosis in this group of patients [50]. Even if the patient survives a hospitalisation, the prolonged stay could result in significant physical deconditioning which could further worsen their frailty status.

Aligning care with patients’ values and priorities is an integral component of PCC. Appreciating what matters to them and tailoring care will promote patient autonomy and alleviate treatment burden [51]. Management of older patients with HF and frailty should not be limited to hard outcomes such as prevention of hospitalisation or prolongation of survival. Rather, it should also aim to enhance functional independence and QoL. The consistency of our study findings with current literature lends support to the utility of patient-reported outcomes in this cohort of patients [21, 52, 53], alongside the traditional measures.

### Strengths & limitations

To the best of our knowledge, this is the first study to comprehensively understand the healthcare needs and goals of older people with HF and frailty, from the perspectives of three different groups: patients, carers and clinicians. We utilised a mixture of qualitative methods (i.e., surveys and interviews) and recruited a large sample size to ensure robustness of the results. We prioritised involvement of ethnically diverse population in order to understand the influence of cultural differences on the needs of these patients.

This study has several limitations. Our study focused on the needs of older adults aged ≥ 65 years old. The results may not be generalisable to younger patients living with HF and/ or frailty. It should be recognised that frailty exists at younger ages [54] and that future studies could explore interventions for younger people living with frailty who have HF.

Our patient sample predominantly comprised of CFS 5 (mild) and CFS 6 (moderate) class of frailty. Only three participants were of CFS 7, none of CFS 8 or 9. Despite our best efforts, representation from the more advanced frailty categories was limited as these patients were mostly house-bound receiving care at the community setting. A few patients (of CFS 7) were unable to participate in the study in view of cognitive impairment and consequent inability to provide informed consent. Nevertheless, our engagement of their accompanying carers enabled exploration of their health care needs, albeit from carer’s lens.

For participants with language barrier, interpreters were engaged to facilitate communication. It is plausible that some essence of our participants’ responses could have been lost in translation. Similarly, survey responses that were transcribed and summarised by the researchers on behalf of participants with reduced dexterity could have resulted in simplified answers. To mitigate the risk of decontexualisation, all survey responses were analysed contemporaneously with the field notes documented during data collection.

A comparison of responses between the different participant groups (i.e., patients, carers and clinicians) would have added depth to the discussion. However, a meaningful analysis was limited by the scope and nature of the current dataset.

## Conclusion

Older patients living with HF and frailty are a complex clinical cohort and understanding of their specific healthcare needs is imperative in the delivery of effective person- centred care. Findings from our study highlight the heterogeneity of their needs, necessitating a multidisciplinary approach when caring for this patient population. Preservation of physical function emerged as an important health-related goal for these patients. Recognition of their individual health priorities is essential for provision of a meaningful goal-concordant care. Future research should focus on reviewing the current care pathway for older people with HF and frailty, and prioritising initiatives towards development of a holistic model of care, incorporating multidisciplinary team to ensure delivery of comprehensive care to this population.

## Supplementary Information

Supplementary Material 1.

## Data Availability

The anonymised datasets generated during and/or analysed during the current study are available from the corresponding author on reasonable request.

## Abbreviations

ACP: Advance Care Planning
ADL: Activities of Daily Living
CFS: Clinical Frailty Score
GDMT: Guideline-directed medical therapy
GP: General Practitioner
HCP: Healthcare Professional
HF: Heart failure
HFrEF: Heart Failure with Reduced Ejection Fraction
HFmRF: Heart Failure with Mildly Reduced Ejection Fraction
HFpEF: Heart Failure with Preserved Ejection Fraction
NHS: National Health Service
PCC: Person- centered Care
QoL: Quality of Life
SRQR: Standards for Reporting Qualitative Research
UK: United Kingdom
