# Supplementary material for: Healthcare needs and priorities of older people living with heart failure and frailty: a multi-perspective study of patients, carers and clinicians
Source: BMC Geriatr. 2026 Jan 7;26:163. doi: 10.1186/s12877-025-06926-1 (PMC12870965; doi:10.1186/s12877-025-06926-1)
Supplement: Supplementary file 1 — Supplementary Material 1. [file 12877_2025_6926_MOESM1_ESM.pdf]

Healthcare needs and priorities of older people living with heart failure and frailty: a multi-perspective study of patients, caregivers and clinicians

Table S1 Essential healthcare needs of older patients with HF and frailty, sub-themes and illustrative quotes from surveys and interviews

Sub-themes	Participants	Illustrative quotes
Management of medical issues	Patients	Like to walk but cannot walk because of breathlessness **(SP30L, Female, 77, CFS 5)**
		Want to walk at least 10 yards without feeling breathless **(SP34L, Male, 82, CFS 6)**
		Leg swelling to go down so I can walk **(SP28L, Female, 88, CFS 6)**
		To have the hip joint issue fixed **(SP19L, Male, 83, CFS 5)**
		All I'm concerned about is my own disease. I mean I'm going through a stage now where I've had, over the last 12 months, I've had rashes all over my body **(ITP1L, Male, 83, CFS 6)**
		It was really bad. How will you feel going (*demonstrates being breathless*)? and I don't do anything! If you'd done some hard work, you could understood it but....**(ITP5L, Male, 87, CFS 5)**
		Breathlessness…could hardly walk upstairs. I had to do 1 step at a time, breathe, 1 step at a time, breathe, 1 step at a time, breathe, 1 step at a time, breathe **(ITP6L, Male, 90, CFS 6)**
		I had it 3 years ago. It's the...whatchamacallit....one of the (valve) is closing up....the aorta. That's closing up and they want to replace it. And I see they were going to do it, but no they didn't and this is the result. It kept coming back again. It's the same thing, the aorta…. same thing, same thing and this is the third time. Same thing. Hopefully it should be the last one. **(ITP9L, Male, 86, CFS 5)**
		Because at the moment, you've got no energy, it's how it's affecting, affecting me **(ITP9L, Male, 86, CFS 5)**
		Ongoing consistent and coordinated support for all conditions **(SP15L, Male, 66, CFS 5)**
	Caregivers	Gets breathless even dressing up, sleeps all the time, fatigued **(SC39L, Male, Carer of CFS 6)**
		Swelling…..she's having some water in the lungs, that's why swelling in face and legs....that's also affecting her day-to-day work **(ITC3L, Female, Carer of CFS 5)**
		Holistic care- attend to all aspects of care apart from heart condition **(SC22L, Male, Carer of CFS 5)**
	Clinicians	Reducing their symptoms (eg breathlessness & pedal oedema), to the point that they are still able to go about their lives & hobbies. **(SH4L, Female, Doctor)**
		Patients often are admitted for an acute illness that is secondary to co-morbidities which are not addressed **(SH13L, Female, Pharmacist)**
		They are all multimorbid, they are not easy to look after and you are not necessarily just looking after their heart failure, their diabetes, their airway disease, anaemia you know all these high output problems as well **(ITH2L, Female, HF Nurse)**
		Advanced care planning, family and patient involvement in recognising when is the time for palliation/ DNAR **(SH3L, Female, Nurse)**

**Management of medical issues**		In terminal/ end stage HF- earlier referral to palliative team; advance care planning- discussion usually left for GP to sort but I believe the cardiology team can facilitate this better **(SH36L, Female, Doctor)**<br><br>I think if you've got that window to discuss palliative care with the patient you should and I don't think all advance care planning should be left for the palliative care team because that seems to happen a lot **(ITH2L, Female, HF Nurse)**<br><br>The other thing that I find really important is about opening up their conversation about advance care planning and doing that at the early stage as possible so people have thought it through and not landing on them at the end, the kind of things like power of attorneys, they've got their wills done and made arrangements for their funeral when death is quite a way off. If you leave it too late then people link it to the fact that they are dying and so I think the earlier you bring it in, you can bring in the reflection that we will all die and I think it can be easier to manage than when you do it quite late and it doesn't get mixed up with the fact that they may be dying. **(ITH4L, Female, Palliative Care Doctor)**<br><br>You ask about breathlessness, you ask about pain, you ask about nausea, you ask about balance and you ask about what their wishes are as it gets towards the end of life. You know just make it routine questioning you know as part of the normal consultation **(ITH4L, Female, Palliative Care Doctor)**<br><br>I always say this, there are risks to not advance care planning and not doing DNR or CPR and those risks are that you'd deprive that person of the good death that they wanted and the quality of life that they wanted at the end of their life **(ITH6L, Female, Geriatrician)**<br><br>So again, (ACP discussion) is a very nuanced thing. It's something that we because we do it all the time and we're very practiced in it. Again, it's a skill. **(ITH6L, Female, Geriatrician)**
**Regain Physical Functioning**	**Patients**	I just want you people to make me walk. I cannot walk. Walking is most important thing in my life. If once I start walking, then I'm alright. I just want to walk **(ITP2L, Male, 72, CFS 6)**<br><br>Frustrating mind you. VERY very frustrating! because you can't do things you wanted to do at the end of the day... it just annoys me that's all it is to me. It annoys me. **(ITP9L, Male, 86, CFS 5)**<br><br>The trouble is I can't stand steady on my feet. I feel a bit weak in my legs. They are not stable enough. It's not the whisky, it's due to my condition. Fell over a few times. I don't know whether it's gone or not but I've got a black eye **(ITP1L, Male, 83, CFS 6)**<br><br>I feel fragile - cannot do anything without feeling strenuous **(SP41L, Male, 83, CFS 6)**<br><br>I slither; legs give way; I wobble - that's my trouble; scared of falling over when walking, so I prefer to use wheelchair **(SP33L, Male, 88, CFS 6)**<br><br>Many falls because I have weak legs; got to watch yourself to stop falling **(SP13L, Male, 72, CFS 6)**<br><br>Have had 9 falls because of loss of balance & giddiness **(SP17L, Male, 72, CFS 6)**<br><br>Last 2 night I had at home, I fell over each night, just walking on a carpet to the bathroom which is in the corner of our suite and I felt like an old fat toad on the flour wobbling about trying to get up and eventually I managed to get up after lot of breathlessness and waiting for it to get better. I'm not sure why I had the fall. It was pretty flat, carpet floor. Nothing was in the way. I was going to the loo. And each time.....I just went. It's rather like I don't remember fainting **(ITP6L, Male, 90, CFS 6)**<br><br>Like gardening but not very active anymore; not able to use treadmill anymore **(SP19L, Male, 83, CFS 5)**<br><br>Enjoys travelling but limited now by breathlessness/ tiredness; misses walking **(SP21L, Female, 77, CFS 5)**

**Regain Physical Functioning**		Play golf as before without feeling breathless **(SP11L, Male, 81, CFS 5)**<br><br>I still do a lot like clay pigeon shooting. I do that tremendous but since I had this you know, very bad, it's really top class shooting but I've not been since I've got this. But by all means I should start once I know I've got control of it **(ITP5L, Male, 87, CFS 5)**<br><br>Doesn't want to be in hospital - I only sleep, sleep, sleep here (**SP68L, Female, 83, CFS 5)**
	**Caregivers**	More rehab classes/ clinics **(SC8L, Male, Carer of CFS 5)**<br><br>Lost confidence to walk, always in fear of falling over **(SC12L, Male, Carer of CFS 6)**<br><br>Being able to walk/ move about without fear of falling; petrified of fall **(SC17L, Female, Carer of CFS 6)**<br><br>More motivation to participate in physical activities **(SC17L, Female, Carer of CFS 6)**<br><br>Used to be able to do a lot of things together (eg travelling) but not now due to breathlessness, limits her ability to go out **(SC22L, Male, Carer of CFS 5)**
	**Clinicians**	Improving patient's mobility to the same level pre-admission **(SH1L, Female, Occupational Therapist)**<br><br>Independence being promoted during hospital stay; independence being promoted by all health professionals to prevent deconditioning; increased risk of deconditioning in hospital and therefore many need increased care such as package of care on discharge **(SH26L, Female, Occupational Therapist)**<br><br>Patients are often left in bed for long periods of time while receiving treatment leading to poorer outcomes around mobility, independence, strength, stamina, pressure sores **(SH28L, Female, Occupational Therapist)**<br><br>Rehabilitation to maintain fitness & regain fitness & mobility following an exacerbation **(SH46L, Female, Doctor)**<br><br>If/when they do require admission which invariably most do, they become significantly deconditioned at which point their frailty becomes the primary limiting factor to be discharged/ improve. In this instance their need would shift to preventing deconditioning which is not properly done in wards/ non-geriatric specialist setting **(SH36L, Female, Doctor)**<br><br>One of the biggest challenges is to get these frail patients moving. Because HF patients they have to keep moving. They can't stop and lie down and be sedentary. But frailty is one of the causes of immobilisation. So it's just a vicious cycle from there, where do you stop it? how do you make frail patients move? Availability of rehab is not very good here. Patient take very long time to be referred to rehab care, which I think should be improved. If frail patients have good access to rehab facilities, then I think it will improve their condition much better. It helps with their overall care, not just drugs. **(ITH11L, Male, HF Doctor)**<br><br>There's nothing really currently in place for really frail patients who don't fit the criteria for cardiac rehab **(ITH8L, Female, HF Nurse)**
**Pharmaceutical Care**	**Patients**	I get fed up with the medications, too many tablets **(SP66L, Male, 82, CFS 5)**<br><br>Tonnes of medications, don't think my body can take all the medications **(SP34L, Male, 82, CFS 6)**<br><br>I take quite a few (medications)...enough to make me rattle! **(ITP4L, Female, 86, CFS 6)**<br><br>A lot of medications; confused about medication titration **(SP13L, Male, 72, CFS 6)**

**Pharmaceutical Care**		Too many pills; if all can be in 1 pill; medication instructions in Gujarati so I can read **(SP21L, Female, 77, CFS 5)**<br>Don't like iron tablets (teeth discoloration & constipation) **(SP5L, Female, 80, CFS 5)**<br>I take 4 Paracetamol and I don't know what the others are for. It's what the doctors prescribe. And that's what I take. I take 5 in the evening, and 4 in the morning. **(ITP5L, Male, 87, CFS 5)**<br>Now Dr XXX wants to get rid of (isosorbide) mononitrate. I've been using that since 2003. And suddenly if that disappears, what impact would that have on my heart? They didn't explain to me. The explanation is so important. Now, doubts in my mind. Communication is very important and put people at ease. **(ITP11L, Male, 79, CFS 5)**<br>To explain what each medication is for **(SP17L, Male, 72, CFS 6)**
	**Caregivers**	Better understanding of medications and why they are taking it **(SC5L, Female, Carer of CFS 5)**<br>Difficulty remembering to take medications **(SC14L, Female, Carer of CFS 6)**<br>Less frequent changes of tablets; prefers to stay on the same tablets and when tablets are changed, it throws him off **(SC27L, Female, Carer of CFS 6)**<br>For the carer to be well informed of the instructions on medications **(SC12L, Male, Carer of CFS 6)**<br>Education regarding medication changes **(SC24L, Female, Carer of CFS 6)**<br>Struggling to swallow medications **(SC42L, Male, Carer of CFS 6)**<br>He's been on some water tablets…they make him go to the loo quite a bit **(ITC4L, Female, Carer of 5)**<br>Lack of sleep (due to taking loop diuretic at night), daytime sleepiness **(SC7L, Female, Carer of CFS 7)**<br>When we are going for shopping, she wants to come with me and that time very difficult for her (because she's on diuretics). If no toilet found then she can't hold. It's very difficult. So sometimes I use pads **(ITC3L, Female, Carer of 5)**<br>I think with one of them is for urine so she does complaint that she need to urinate a lot but that's the side effects right? **(ITC5L, Male, Carer of 6)**
	**Clinicians**	Optimising dosing of ACEI & BB; optimising 2nd line therapy: aldosterone antagonist therapy (balancing BP and U&E results) & SGLT2i therapy; knowing when & timely initiating third line therapy (eg Digoxin, Entresto, cardiac resynchronisation therapy) **(SH5L, Female, Doctor)**<br>Continent needs due to diuretics increasing incontinence issues. This also leads to skin integrity issues due to moisture damage; UTIs-frequent UTI due to increased urine production **(SH44L, Female, Nurse)**<br>Recurrent HF admissions due to poor compliance with treatment **(SH24L, Female, Doctor)**<br>Many of my patients tell me once they took the Furosemide, that's it, their day is gone. So if they want to go out to the mall that day they actually have to stop taking the Furosemide and later on they have to pay the consequences. **(ITH11L, Male, HF Doctor)**<br>A case in hand is a patient that I saw last week, lives alone doesn't need a package of care, is independent with things but fine…her diuretics…I mean she's hugely overloaded…but diuretic therapy she just won't take it or she won't take it consistently because she needs to go outdoors shopping, it's that that I struggle with, with some of the patients, with their adherence to medications that we suggest. It prevents them from being able to have their social inclusion because they can't go out when they are taking…I mean she's on 120mg twice

**Pharmaceutical Care**		daily Furosemide and she just said I don't take it some days because I've got day centres, I've got things that I want to go and do, I've got shopping and it just impacts on their lives doesn't it? **(ITH2L, Female, HF Nurse)**<br><br>Weighing the risk and benefit of treatment to these patients as you know they are very prone to the adverse effects of the drugs that we give although we know that the benefits are very good. But also they come with very risky adverse event like hypotension that can lead to syncopal attack. And you know that if frail patients fall, then it's downhill from there on **(ITH11L, Male, HF Doctor)**<br><br>Optimal medication management; rationalising medication- polypharmacy can become very complex & cumbersome; increased urinary frequency due to diuretic therapy - frustrating & can increase risk of falls **(SH5L, Female, Doctor)**<br><br>The other thing I haven't mentioned about is rationalising medications. He might not be able to take many off but I'd say any medication that can come off….you know just thinking about what are the benefits of these? What are the number needed to treat? And are they so high that the likelihood of this person in front of me now is benefitting from the treatment is probably not worth him taking that tablet everyday. **(ITH4L, Female, Palliative Care Doctor)**<br><br>In general I can see that the association between failure and frailty sometime is lost. Yes, sometime, especially on the wards, they concentrate too much about - *Oh, it's heart failure, with reduced ejection fraction, we have LVSD so we need all the drugs*. *All together*. *So we need to start everything*. *We need to start Entresto.* Not thinking about also the best interests of the patient. Is it really beneficial? If they're going to fall the next day? And they're going to experience severe dizziness from this drug and their quality of life will be impacted anyway. Of course, on the other end we need to make sure that we actually managing heart failure. So, it depends on the case, of course, of the patient. **(ITH9L, Female, HF Nurse)**<br><br>I think we just carry on with things and we don't really question and this is like my mum's on a medication for something 25 years ago and no one has really thought about it and she carries on taking it and you're like "*why you're on it*?" and mum's like "*I don't know!*" (laughing)…maybe we can think about stopping that? **(ITH4L, Female, Palliative Care Doctor)**<br><br>You don't have access to all that information because you know that patients coming in don't have all that information. "*Why are you taking that*? *Oh, I've been taking that for 20 years*. *What you take it for? I don't know, I just keep taking it*". **(ITH5L, Female, Pharmacist)**<br><br>Sometimes it's about reducing that medication burden…they often feel burdened by all these outpatient appointments and medications because again, you build up side effects. You build up anticholinergic burden. **(ITH6L, Female, Geriatrician)**<br><br>Lots of patients don't seem to know what medications they are on for their HF or why. I believe that patient education is important, especially when they are on multiple HF medications **(SH4L, Female, Doctor)**<br><br>My plan would be, I would like to do a full, this is what you're on, this is what you're taking it for, this is how it works. I would like to do the education in there. **(ITH5L, Female, Pharmacist)**<br><br>If they're not feeling symptomatic…..then they won't….they will say. *Why do I need to change anything? Because I'm OK now I'm feeling fine*. *I don't want to do any more changes.* So make sure that they actually understand the reason why it's so important that we're not just doing it because we want to do it. So better education, better information that would be good. **(ITH9L, Female, HF Nurse)**
**Nutritional Care**	**Patients**	No appetite **(SP17L, Male, 72, CFS 6)**<br><br>Mostly ready-made meal **(SP13L, Male, 72, CFS 6)**<br><br>Mostly has frozen food/ microwaved (**SP19L, Male, 83, CFS 5)**<br><br>Eating less these days-I'm off food **(SP40L, Male, 82, CFS 5)**

Nutritional Care		Struggle with swallowing, dry mouth- only boiled food, loss of appetite **(SP71L, Female, 67, CFS 5)**<br>Can't chew because of trigeminal neuralgia **(SP13L, Male, 72, CFS 6)**<br>Eating less due to breathlessness, lost 25kg **(SP84L, Male, 79, CFS 6)**<br>I lost the taste, something like I lost taste of food I was eating. Started to cut down on my food. I did not enjoy eating, I did not. **(ITP3L, Male, 74, CFS 5)**<br>And I was living on my own because my wife had died. I don't think I was eating properly. I'd lost 2 stones in weight, which was unusual for me. And then I noticed, shortly before I came into the Glenfield Hospital that I couldn't taste anything. And mouth always seems to be dry and I was just off food **(ITP6L, Male, 90, CFS 6)**
	**Caregivers**	Before she was eating properly but now because of breathing, she eats less. She can't eat more. But before she eat very good **(ITC3L, Female, Carer of 5)**<br>Poor appetite, need regular prescription for Fortisip from the GP **(SC7L, Female, Carer of CFS 7)**<br>Needs good healthy diet; poor appetite on dietary supplement **(SC10L, Female, Carer of CFS 5)**<br>To improve patient's appetite **(SC39L, Male, Carer of CFS 6)**<br>More education on type of diet, need to rely on online educations (low fat, low salt etc) **(SP40L, Male, Carer of CFS 6)**
	**Clinicians**	Nutrition, poor appetite **(SH2L, Female, Nurse)**<br>Encourage low salt intake **(SH19L, Female, Nurse)**<br>Individualised care to help with their diet… allow them to maintain their desired lifestyle **(SH46L, Female, Doctor)**<br>A lot of education that need to be done but I think it's really difficult to change their habits of people really because they are really set in their ways and it takes a significant event in their lives to actually want to change something like that. **(ITH3L, Male, Dietitian)**<br>I give them the advice regarding the limited salt intake. And then I also emphasise about the pre-packed meals because sometimes some people don't know about, you know, that just because you don't have salt in there doesn't mean there's not enough salt. So just being aware and reading the label. **(ITH8L, Female, HF Nurse)**<br>Salt is tricky….the population...they are 65 and over, a lot of them probably would have ready meals. A lot of them would probably have old style of cooking…oh some salt….you know… **(ITH3L, Male, Dietitian)**
**Assistance with ADL**	**Patients**	Unable to shower- bending down gives chest pain, needs to sit on a stool to shower **(SP41L, Male, 83, CFS 6)**<br>Need to wait for son to come home late evening to help with showering **(SP70L, Male, 84, CFS 6)**<br>Even normal toileting is difficult **(SP20L, Female, 71, CFS 5)**<br>Need a carer who can spend more time caring - 30 minutes/day is not sufficient **(SP28L, Female, 88, CFS 6)**

Assistance with ADL		The carer firm is pretty decent, you know. But it's not very sufficient to carry out everything... but you've got to think of what you can afford to buy for you **(ITP1L, Male, 83, CFS 6)**<br><br>Feels anxious about going home (after discharge), not knowing how to manage cooking **(SP74L, Female, 75, CFS 5)**<br><br>Caters food/tiffin from outside (5 days/ week; £7.50/meal **(SP41L, Male, 83, CFS 6)**<br><br>I live on my own because my wife has died. I buy in frozen 1 person meals from a very good farmer shop or Waitross or Sainsbury's. And the trouble is, I'm not used to cooking regularly, and my wife, not only was a very good cook but she also a dietitian until we were married. So she keep me right with the food. We've got to consider what's going to happen in the future when I go home (after discharge) **(ITP6L, Male, 90, CFS 6)**<br><br>My husband he's become senile. And for 4 days he kept forgetting to get my medications. I'm disabled. It made me very ill. My husband used to (take care of me before) but he can't now. Because he's ever so forgetful. He's going to be sorted out this afternoon. **(ITP4L, Female, 86, CFS 6)**<br><br>My 2 granddaughters are going to come take the dog out and come and do some jobs for us. And I think they are going to get some carer to help cleaning. I couldn't do it (cleaning) but (husband) could. Now he can't. **(ITP4L, Female, 86, CFS 6)**
	Caregivers	Physical support for moving around **(SC2L, Male, Carer of CFS 6)**<br><br>Help with maintaining personal hygiene care, tasks around the house, easy access to home mobility aids **(SC17L, Female, Carer of CFS 6)**<br><br>Assistance with daily activities eg. cooking, cleaning, bathing **(SC19L, Male, Carer of CFS 5)**<br><br>People to look after him at home, needs help with personal care, food, clean clothes, breathless - unable to do most things these days **(SC28L, Female, Carer of CFS 6)**<br><br>I could do without look after someone but then you cannot get anybody when you want them **(ITC1L, Male, Carer of 6)**<br><br>My trouble is remembering… my brain is out; she's losing her memory as well but don't tell her that. It upsets her.... **(ITC1L, Male, Carer of 6)**<br><br>Yes, it is very difficult. Shove the hoover with one hand and hold the (walking) stick with the other because you can't put the weight on an hoover, like you have to put the weight on the stick. But this (knee) joint…I've got no joint here….that's out of socket there **(ITC1L, Male, Carer of 6)**<br><br>So washing, dressing, cooking, medications, everything…breakfast everything. Housework, shopping as well. I have to take her (into shower). Go with her all the time **(ITC3L, Female, Carer of 5)**
	Clinicians	Confidence to do day-to-day tasks independently and safely **(SHL1, Female, Occupational Therapist)**<br><br>Social input is particularly important for those patients with frailty, potentially needing package of care/ nursing home placements on discharge **(SH4L, Female, Doctor)**<br><br>Being able to manage their activities of daily living and remain independent at home **(SH14L, Female, Physiotherapist)**<br><br>Physiotherapy and occupational therapy to optimise independence at home **(SH31L, Female, Doctor)**

**Assistance with ADL**		The other need that often comes out is social support and social services, and there's also carer burden, carer strain and carer stress. Sometimes when we do our front door frailty assessments or do our community geriatrics, we link in with things like social services support and try and deliver on those things as well for patients because that can be actually sometimes more important than the physical health or the medical need **(ITH6L, Female, Geriatrician)**<br><br>I found, the ones who have relatives with them…. they have their help at home from their next of kin or not only at home, just in general. They are more assisted in general. Otherwise, patient that normally lives alone, they're left…they are not provided **(ITH9L, Female, HF Nurse)**
**Environmental & Social Support**	**Patients**	Patient slipped and fell in the toilet because nothing to hold on to **(SP33L, Male, 88, CFS 6)**<br><br>Climb stairs like cat or dog **(SP74L, Female, 75, CFS 5)**<br><br>I moved around with the furniture (at home) **(ITP4L, Female, 86, CFS 6)**<br><br>Daughter/ son are busy with work unable to provide care, feels very lonely and upset; needs help with home adaptation, walk-in shower, don't know where to get help **(SP77L, Female, 67, CFS 6)**<br><br>It is not a criticism but an issue to be noted. There is a lack of coordination. One group is very humane, very professional, very communicative. Not just one person, one group. The other group, they just want to chop...chop....chop...factory type, as if there is a pressure from the top. They want to get done. They want to empty the beds. "*Right, you can go home*. *You can go home"*. Now that doesn't give heart to the heart patient. It demoralises you, because of the emotional impact to the heart patient can be severe **(ITP11L, Male, 79, CFS 5)**<br><br>Oh yeah I don't have to suffer on my own. I've only got to mention my name and that's it. They (social support group) will come around. **(ITP1L, Male, 83, CFS 6)**<br><br>And I have a great support of my wife who was also meticulous and my 2 daughters who are 60 and 58…as s a team, we are a team **(ITP6L, Male, 90, CFS 6)**<br><br>You see I used to walk a lot. Me and my wife. Since she (died) I don't walk much at all. And it started creeping in bit by bit. And you don't really know it. Because you got other things to do and to get over. **(ITP9L, Male, 86, CFS 5)**
**Environmental & Social Support**	**Caregivers**	Yeah, railing. I have already applied for re-housing but I'm in waiting list now. So because of my father-in-law, he's a major paralysis, he can't walk a single step. So they put us in Band 1 but still we have to wait....1 year. Because their room is upstairs, bedroom is upstairs and it's very difficult for me to manage everything… breakfast, cooking downstairs and bringing everything upstairs, laundry, everything, It's very hard for me **(ITC3L, Female, Carer of 5)**<br><br>It would be nice if there was a group of people that regularly visited for a chat. Its a long day when you're poorly and can't do much, there is only so much conversation a wife can offer **(SC21L, Female, Carer of CFS 6)**<br><br>Going out into the community as socially isolated, more contact from community-based services **(SC27L, Female, Carer of CFS 6)**<br><br>Possibility of getting in touch with someone when help is needed in a timely manner, prefer contact with more people (e.g., support group) to increase awareness and experience of own condition **(SC29L, Female, Carer of CFS 6)**<br><br>Older people are not patronised by assuming fragility is accompanies by lack of cognitive ability **(SC15L, Female, Carer of CFS 5)**

		To treat them like any other person **(SC3L, Female, Carer of CFS 6)**<br>Company, mental health improvement, social support **(SC10L, Female, Carer of CFS 5)**<br>Compassion and empathy, better understanding of people with disability **(SC37L, Female, Carer of CFS 6)**<br>Obviously as he's getting older I think he had the heart condition for a long time and he's 85 plus, and he lost his wife a few months ago and it has got to take a little bit toll on your health. (**ITC2L, Male, Carer of 6**)
**Environmental & Social Support**	**Clinicians**	Emotional/ psychological impact of living with a chronic illness, fear, frightened **(SH3L, Female, Nurse)**<br>Encourage self-esteem; community support eg HF specialist nurses, peer group support, meeting with other individuals with the condition **(SH19L, Female, Nurse)**<br>Self-esteem and emotional support. Some patients do not take diuretics because they are ashamed wetting themselves, this may lead to low self-esteem. Some may stop taking diuretics to save themselves from embarrassment **(SH19L, Female, Nurse)**<br>Support with new diagnosis, psychologically and emotionally **(SH47L, Female, Nurse)**<br>Their healthcare needs are usually, some of it is social inclusion **(ITH2L, Female, HF Nurse)**<br>The thing I feel is, I think they need more in terms of emotional support as well. I definitely feel the patients need a lot more emotional support. I get on the phone to talk about one thing and patients are telling me all about their, you know, everything that's been going on and they kind of use that time to offload. You know because they have someone to talk to them that will listen to. It's just really listening sometimes, it's not really to answer the question, but to listen to what they have to say. And then you feel they feel better at the end. You know that there's someone there to listen to what they have to say and help support them in terms of their health. **(ITH8L, Female, HF Nurse)**<br>They can also be quite isolated sometimes on their own. And then with their frailty, they can't really go out and search for this information or try and pursue it themselves like an independent person would. So then they just end up just being by themselves and feeling left alone really, to deal with this condition. **(ITH8L, Female, HF Nurse)**<br>They struggle with the stairs…the bedroom is upstairs…the bathroom is downstairs. They fall. It's when they come to hospital. **(ITH9L, Female, HF Nurse)**
**Access to Healthcare**	**Patients**	2 years cannot see GP! **(SP68L, Female, 83, CFS 5)**<br>Getting appointment with GP is a problem, want better communication between GP **(SP20L, Female, 71, CFS 5)**<br>GP practice is not nearly as good as it used to be 10 years ago **(SP34L, Male, 82, CFS 6)**<br>Local surgery is absolutely pathetic! **(SP36L, Female, 76, CFS 5)**<br>3 weeks to see GP for shortness of breath, extremely frustrating **(SP42L, Male, 76, CFS 6)**

**Access to Healthcare**	**Caregivers**	A lot of people have left that surgery because they say they can't get an appointment and when we do get an appointment, the receptionist says the doctor will ring you and sometimes it's 4 hours, 6 hours and you're concerned if the doctor is going to ring me or not **(ITC2L, Male, Carer of 6)**
**Access to Healthcare**	**Caregivers**	Proper follow up, medication to be given regularly, otherwise it is 'mentally stressful' worried about patient's condition **(SC4L, Male, Carer of CFS 5)**
**Access to Healthcare**	**Caregivers**	Regular appointment and check-up, easy access to the clinic for information; possibility of community-based clinics **(SC5L, Female, Carer of CFS 6)**
**Access to Healthcare**	**Caregivers**	Home care if the patient lives alone with consultations/ sharing information with the local GP **(SC6L, Male, Carer of CFS 5)**
**Access to Healthcare**	**Caregivers**	Healthcare is very disjointed - GP doesn't know what medications hospital gives, hospital doesn't know the medications GP gives, it's frustrating **(SC18L, Female, Carer of CFS 7)**
**Access to Healthcare**	**Caregivers**	Regular monitoring of their condition and medication by a specialist nurse/ doctor. I have found my mum's GP was only answering my mum's concerns by phone rather than face to face, having a personalised care plan, a proper pathway of care **(SC20L, Female, Carer of CFS 7)**
**Access to Healthcare**	**Caregivers**	How to escalate for support when needed; clear contact points when concerns rises/ need access for specialist advice **(SC24L, Female, Carer of CFS 6)**
**Access to Healthcare**	**Caregivers**	Don't know what to do or who to contact, so called the ambulance **(SC29L Female, Carer of CFS 6)**
**Access to Healthcare**	**Caregivers**	Knowing what to do when they feel unwell and have confidence calling 999, the long wait time (although understandable) can put patient off **(SC34L, Female, Carer of CFS 6)**
**Access to Healthcare**	**Caregivers**	Community HF nurse - as contact point to contact when needed **(SC41L, Female, Carer of CFS 5)**
**Access to Healthcare**	**Caregivers**	I'd say "*no I go ring the doctor*" and she (patient) won't let me ring the doctor. But until she struggles to get by and she finds out she's got to do because there's nothing else you can do for it. But if somebody comes every…once a month and find out before I can…. It probably might be a good idea if somebody could come to see her at times, once a month and they know what's happening, with each trouble she's having **(ITC1L, Male, Carer of 6)**
**Access to Healthcare**	**Clinicians**	Regular follow up by community HF/ GP to try and prevent worsening HF/ need to be admitted **(SH3L, Female, Nurse)**
**Access to Healthcare**	**Clinicians**	Community HF team to look after them holistically when at home **(SH22L, Male, Doctor)**
**Access to Healthcare**	**Clinicians**	Acute changes in symptoms- not addressed in timely/ appropriate time frame in community (GP) **(SH41L, Female, Pharmacist)**
**Access to Healthcare**	**Clinicians**	Care level drops post discharge; early accessibility to GP care for even mild/early symptom may help recognise/ prevent lot of admissions due to infections & other causes **(SH37L, Male, Doctor)**
**Access to Healthcare**	**Clinicians**	Their queries can be dealt with and they can be managed at home rather than having to get worse and then be readmitted into hospital. Or struggle to get appointments with the GP and then that takes a long process. **(ITH8L, Female, HF Nurse)**
**Access to Healthcare**	**Clinicians**	The minimum they should be seen by a treating physician is at least 6 months apart even if they are stable because no HF is actually stable. You already have HF so means you know it's a myth saying that… we stop labelling patients as stable HF because HF is never a stable disease, so you don't know when they are going to deteriorate and it's important that you pick up early signs even during casual clinic follow up you might pick up something like sleep apnoea, they might forget to ask that beforehand so you can manage that better and

**Access to Healthcare**		having like minimum number of follow up/ year. In my opinion, at least 6 months if the patient is totally, totally symptoms free so that everything is in place. **(ITH11L, Male, HF Doctor)**<br><br>They could have like appointment one here and appointment two there and then it takes 2 hours to get there and back. There is no coordination. And we don't also question do they need to come? I think there is a role for a person to coordinate especially if they get more complicated and they are under 5 or 6 different specialties **(ITH4L, Female, Palliative Care Doctor)**<br><br>Having one person they can contact about their HF like a "key worker" **(SH14L, Female, Physiotherapist)**<br><br>And so ideally the geriatrician would or the whoever is a gatekeeper or the care coordinator would liaise with the rest of the team and bring that team together. So there's something about as well as reducing burden, it's also about bringing other expertise and specialty into the patients care. **(ITH6L, Female, Geriatrician)**<br><br>Some of these patients also struggle to actually get to GP… these frailer patients. So like I was saying, they might leave things off later because the GPs sometimes are reluctant to come home. So you know, that's an extra step for a home GP visit rather than trying to get an appointment within the actual practise. So some of these patients might be missed and then it ends up to the point where they have to come into hospital. **(ITH8L, Female, HF Nurse)**

Table S2 Perceived health-related goals of older people with HF and frailty, sub-themes and illustrative quotes from surveys and interviews

Sub-themes	Participants	Illustrative quotes
ADL-Independent	Patients	My independence **(SP20L, Female, 71, CFS 5)**<br><br>To deal with things on your own, be in control of things **(SP12L, Female, 79, CFS 5)**<br><br>Now one thing I like to be is self-sufficient. My independence is important for me. It's not just for me but also gives confidence to my wife. We've been together for 52 years. So that is something important **(ITP11L, Male, 79, CFS 5)**<br><br>I live on myself. I lost my wife 3 years ago, no 4 years now. She had dementia. So I do my own thing for myself and the house. I don't have any one come in. I do my own cleaning. I do my own cooking, yeah sort of improved….I eat it anyway. Washing up and what not. That's how I've been brought up. My own shopping, cleaning, the usual things we all do and that keeps me busy. **(ITP9L, Male, 86, CFS 5)**<br><br>My granddaughter's husband is a football coach…they live in Dubai, but he's said he's going to send a few strengthening muscle…muscle strengthening exercises because he knows, they come over every so often, and he knows how I'm struggling with walking about… anything that's going to strengthen the muscles in my legs **(ITP7L, Female, 75, CFS 5)**
	Caregivers	Independence, he likes to do things on his own **(SC28L, Female, Carer of CFS 6)**<br><br>To look after themselves; to have control over things on her own **(SC16L, Male, Carer of CFS 5)**<br><br>Having support to stay as independent as possible **(SC20L, Female, Carer of CFS 7)**<br><br>Remain independent as possible, be able to communicate their wishes, continue to have dignity, respect, remain mobile as possible **(SC27L, Female, Carer of CFS 6)**<br><br>But he wants to do everything he can, which is a good thing, positive thing. When you want to do things you feel positive you don't want to give up **(ITC2L, Male, Carer of 6)**<br><br>Well, he likes to go back to his old self. He's very independent anyway. **(ITC4L, Female, Carer of 5)**
	Clinicians	Independence; being able to manage task that are important to them as independently as possible **(SH26L, Female, Occupational Therapist)**<br><br>Most patients raise concern about their daily living activities **(SH10L, Female, Nurse)**<br><br>Functional ability i.e., ability to maintain independence/ complete ADLs unassisted **(SH32L, Male, Doctor)**<br><br>Being able to manage their activities of daily living and remain independent at home **(SH14L, Female, Physiotherapist)**<br><br>Good cardiac rehab programme to maintain fitness/ avoid deconditioning **(SH18L, Male, Doctor)**<br><br>Regaining as much independence as possible (eg cleaning, cooking, caring for self) **(SH21L, Female, Doctor)**<br><br>Because actually, when you speak to most people, most of the time, their greatest fear is being very dependent and ending up in a nursing home. They've always said *"I don't want to be a nursing home. I don't want to be dependent on people. That's my worst…one of my worst fears*". **(ITH6L, Female, Geriatrician)**

**Remaining symptom-free**	**Patients**	To walk without feeling breathless and fatigue **(SP20L, Female, 71, CFS 5)**<br>Not wake up at night feeling breathless **(SP19L, Male, 83, CFS 5)**<br>Leg swelling to go down so she can walk, "*love to walk*", "want to keep going" **(SP28L, Female, 88, CFS 6)**<br>Loves to do gardening but unable to do as much because of breathlessness; "*I'm a wreck now*" **(SP32L, Female, 84, CFS 6)**<br>I was (having trouble going upstairs). That's when I went to the doctors. But I feel, I tell ya, and I know this… I'm a lot better because I know where I was with my breathing. And now it seems to clear and I'm not (*demonstrates panting*) that... **(ITP5L, Male, 87, CFS 5)**
	**Caregivers**	As mobile as possible- gets short of breath walking from 1 room to another **(SC10L, Female, Carer of CFS 5)**<br>Being able to walk 15 yards without feeling short of breath **(SC13L, Female, Carer of CFS 5)**<br>Get back to normality, get rid of breathlessness **(SC22L, Male, Carer of CFS 5)**<br>Awareness about symptom management **(SC32L, Male, Carer of CFS 5)**
	**Clinicians**	Symptoms (particularly breathlessness, oedema) are controlled **(SH3L, Female, Nurse)**<br>Reducing their symptoms (eg breathlessness & pedal oedema), to the point that they are still able to go about their lives & hobbies. Important to ensure that these patients are comfortable & not in too much stress/ burden by their HF (eg breathlessness) **(SH4L, Female, Doctor)**<br>Symptom management and effects on their daily life including how this affects their basic ADLs and ability to engage in hobbies **(SH25L, Female, Doctor)**<br>Symptom improvement, shortness of breath, constant tiredness & fatigue **(SH35L, Female, Pharmacist)**<br>Being able to do simple tasks such as dressing themselves/ walking without being breathless **(SH48L, Female, Healthcare Attendant)**
**Improved QoL**	**Patients**	To have good quality of life and look after myself **(SP36L, Female, 76, CFS 5)**<br>To be happy! Yeah as happy as I can. Not worried. I mean worry can kill years of anybody. **(ITP5L, Male, 87, CFS 5)**<br>To feel better again in my life; used to be active all the years, wake up at 4am to go to gym and walk, swim 30 laps; recent health has greatly limited ADLs **(SP22L, Male, 74, CFS 5)**<br>To stay fit, looking after (his) pets and gardening **(SP25L, Male, 89, CFS 5)**<br>"Health is wealth"; to stay active and stay strong **(SP27L, Male, 92, CFS 5)**<br>Apprehensive about things going wrong due to shortness of breath; "scared of getting out of the house" due to shortness of breath **(SP45L, Female, 86, CFS 5)**<br>To go to temple as frequently as possible (now unable due to health/ breathlessness) **(SP47L, Female, 86, CFS 5)**

Improved QoL		Would like to go for walking as much as possible if can, "but I can't now" because of breathless and tiredness; feels QoL is reduced **(SP48L, Male, 88, CFS 5)**<br>I can't just sit here and just carry on because my body is not used to lying around doing nothing. I'm used to working. I've got 3 gardens to look after. I enjoy it, they relax me. Nothing fancy. Mine, my next door neighbour's and the one across the road. **(ITP9L, Male, 86, CFS 5)**
	Caregivers	Retaining independence and QoL **(SC11L, Female, Carer of CFS 5)**<br>Heart specialist and doctors communication with caregivers, correct medication and checks on how to improve QoL **(SC19L, Male, Carer of CFS 5)**
	Clinicians	Improving QoL **(SH8L, Male, Doctor)**<br>To have a QoL and keep as independent as possible **(SH16L, Female, Occupational Therapist)**<br>Their needs to be listened to and to have a good QoL **(SH17L, Female, Nurse)**<br>Understanding priorities of patient and not assessing; ensuring QoL is focus of decision making **(SH43L, Female, Doctor)**<br>I would say from a patient perspective, overall it's about your quality of life isn't it? If your breathlessness is improved, that's great but if your quality of life hasn't improved because of it, you're still living alone and nobody visits. You feel less breathless going to the toilet but it doesn't add significant meaning to your life…that's the point isn't it….what we are trying to do is enable people to live better and not necessarily manage their symptoms, we manage their symptoms so they can live **(ITH4L, Female, Palliative Care Doctor)**<br>Geriatrics is very much patient-centered and focused on quality probably more so really than quantity of life because our patients are approaching the end of their life. So we want to focus on what is going to give benefit to the patients. **(ITH6L, Female, Geriatrician)**
Avoiding Hospitalisation	Patients	Don't like to be in hospital **(SP17L, Male, 72, CFS 6)**<br>Feel fed up coming to hospital because just sitting down all day and not doing anything **(SP21L, Female, 77, CFS 5)**<br>Wants to avoid coming to hospital **(SP42L, Male, 76, CFS 6)**<br>Has had 2-3 hospitalisations, don't feel on top of the world **(SP54L, Female, 83, CFS 6)**<br>Prefers to stay at home because can communicate with other people, feels isolated at the hospital with no one to talk to; language barrier in the hospital -"*everyone speaks English, don't understand*" **(SP67L, Male, 75, CFS 6)**<br>Prefers staying at home, doesn't want to be in hospital - feels self de-conditiong in hospital "I only sleep, sleep, sleep here" **(SP68L, Female, 83, CFS 5)**<br>Be healthy enough to stay out of hospital **(SP69L, Female, 77, CFS 5)**<br>Patient does not like hospital- had episodes of delirium in the past 1 week since admission, prefers to stay at home, in his familiar environment doing his routine things **(SP72L, Male, 87, CFS 7)**
	Caregivers	Staying out of hospital **(SC41L, Female, Carer of CFS 5)**

		Being well and staying at home **(SC20L, Female, Carer of CFS 7)**<br>GP to do home visits; support from GP and community team **(SC18L, Female, Carer of CFS 7)**<br>At home care- to avoid hospital unless really critical **(SC34L, Female, Carer of CFS 6)**
**Avoiding Hospitalisation**	**Clinicians**	Being able to manage symptoms as best as possible in the community/ at home without need for any unnecessary admissions **(SH25L, Female, Doctor)**<br>I think that lots of these patients really value a quick turn around in hospital, they often do not wish to remain in hospital very long **(SH4L, Female, Doctor)**<br>Avoiding multiple repeat hospital admissions; follow up including timely repeat BNP/ echocardiogram/ symptom reviews so any medication adjustments can ideally be made in the community to avoid hospital admissions **(SH5L, Female, Doctor)**<br>Ensure the patient is regularly supported by the NHS (physiotherapy, occupational therapy) so that the patient risk of being admitted for something that can be avoided is low **(SH13L, Female, Pharmacist)**<br>To have their condition managed at home and stay out of hospital **(SH16L, Female, Occupational Therapist)**<br>Spending more time at home rather than in hospital; some people would rather be managed at home with non-interventional treatment **(SH17L, Female, Nurse)**
**Being with Family**	**Patients**	Family and spending time with grandchildren **(SP12L, Female, 79, CFS 5)**<br>To look after his disabled son, to cook for his son **(SP17L, Male, 72, CFS 6)**<br>Being at home with wife and children and 2 dogs **(SP31L, Male, 76, CFS 5)**<br>To spend time with family- *just my family* **(SP32L, Female, 84, CFS 6)**
	**Caregivers**	Prefers to be with family members; doesn’t like to be around others/ other carers **(SC7L, Female, Carer of CFS 7)**<br>To have people that care about her to be around to help and support **(SC19L, Male, Carer of CFS 5)**<br>To spend more time with family **(SC39L, Male, Carer of CFS 6)**<br>Happier at home with family around **(SC41L, Female, Carer of CFS 5)**<br>My father-in-law, he’s paralysed. He’s all the time in the chair and bed. But they like talking with each other, playing with my baby **(ITC3L, Female, Carer of 5)**<br>She’s here, I come here. She probably wants company, I need my company and she’s my company **(ITC1L, Male, Carer of 6)**
	**Clinicians**	Shorter hospital admissions to be home with loved ones **(SH39L, Female, Doctor)**<br>Maintaining a good QoL surrounded by family and friends **(SH53L, Female, Nurse)**

**Not a burden to family**	**Patients**	To look after oneself otherwise you become a burden to your family members **(SP41L, Male, 83, CFS 6)**<br>Independent - not be a burden to wife **(SP42L, Male, 76, CFS 6)**<br>To be in good health to be able to do things, mobility, general health-not a burden to others **(SP52L, Male, 66, CFS 5)**<br>Worried about my husband - he has looked after me for more than 40 years, 24/7 **(SP71L, Female, 67, CFS 5)**<br>Patient feels bad for husband and children, being burdensome to family **(SP74L, Female, 75, CFS 5)**<br>I want to feel useful to them. You know what I mean… I don't want to be, oh, just grandma sat in the corner not doing anything **(ITP7L, Female, 75, CFS 5)**
	**Clinicians**	Regaining as much independence as possible (eg cleaning, cooking, caring for self); not being a burden to family **(SH21L, Female, Doctor)**
**Survival**	**Patients**	To have a long life **(SP31L, Male, 76, CFS 5)**<br>Keeping alive for my family **(SP48L, Male, 88, CFS 5)**<br>That I live, that I get better, that I recover **(SP49L, Male, 65, CFS 5)**<br>To live longer to see grandchildren and great grandchildren **(SP69L, Female,77, CFS 5)**
	**Caregivers**	To be just independent- he can live longer **(SC39L, Male, Carer of CFS 6)**
	**Clinicians**	Reassurance they are being offered all that meets their needs to improve their life expectancy **(SH49L, Female, Advance Healthcare Assistant)**
